# Rural area in a European country from a health care point of view: an adaption of the Rural Ranking Scale

**DOI:** 10.1186/1472-6963-14-147

**Published:** 2014-04-02

**Authors:** Jost Steinhaeuser, Petra Otto, Katja Goetz, Joachim Szecsenyi, Stefanie Joos

**Affiliations:** 1 Department of General Practice and Health Services Research, University Hospital Heidelberg, Vossstrasse 2, Heidelberg 69115, Germany; 2 Competence Centre General Practice Baden-Wuerttemberg, Vossstrasse 2, Heidelberg 69115, Germany

**Keywords:** Family medicine, Health service research, Knowledge transfer, Physician shortage, Rural area

## Abstract

**Background:**

In many countries, rural areas are facing a shortage of general practitioners (GPs). Appropriate strategies to address this challenge are needed. From a health care delivery point of view, the term rural area is often poorly defined. However rural areas have to be adequately defined to ensure specific strategies are tailored to these environments. The aims of this study were to translate the New Zealand 6-item Rural Ranking Scale (RRS), to culturally adapt it and to implement it to identify rural areas from a health care delivery perspective. Therefore we aimed to validate the RRS by defining cut-off scores for urban, semi-rural and rural areas in Germany.

**Methods:**

After receiving permission, two researchers independently translated the RRS. In a consensus meeting, four items were identified that had to be culturally adapted. The modified RRS-Germany (mRRS-G) was sent to 724 GPs located in urban, semi-rural and rural areas to validate the “rurality” scoring system for conditions in Germany.

**Results:**

Four items, “travelling time to next major hospital”, “on-call duty”, “regular peripheral clinic” and “on-call for major traumas” had to be adapted due to differences in the health care system. The survey had a response rate of 33.7%. A factor analysis showed a three dimensional structure of the mRRS-G scale with a poor internal consistency. Nevertheless, the three items regarding “on-call duty”, “next major hospital” and “most distant boundary covered by your practice” were identified as significant predictors for rurality. The adapted cut-off point for rurality in Germany was 16. From this study’s participants, 9 met the RRS cut-off point for rurality (a score of 35 or more).

**Conclusion:**

Compared with New Zealand rurality scores based on this tool, German scores are far less rural from a health care delivery point of view. We consider that the construct of rurality has more aspects than those assessed by the mRRS-G. Nevertheless, rural areas from a health care delivery viewpoint can be effectively defined using mRRS-G and therefore it can support tailored strategies against GPs shortage.

## Background

Many countries face a shortage of general practitioners (GPs) especially in rural areas [1,2]. Therefore concern is growing that health care systems will not be able to provide sufficient, close to home care to meet the future needs of an increasingly aging society [3]. Strategies to face this shortage can be classified as normative-, utilitarian- and coercive strategies [4]. In terms of recruitment or managing shortages, an example of a coercive strategy would be restricting the entry to a particular health care delivery area. Examples of utilitarian strategies are scholarships or educational loan repayments in return for service in underserved areas. Normative strategies include educational and other programs dedicated to the training of doctors willing to work in underserved areas [4].

To identify which national regions in a country might be in need of one or more of such strategies, it is crucial to define “rural” from an individual country point of view. Based on international studies published to date it can be concluded that subjective understandings of what rurality means differ remarkably [5,6]. Approaches to define rurality have come from countries such as Australia, the United Sates of America or Canada, where there is a tradition in rural healthcare research. Depending on the context, rurality has been defined in numerous ways: cost or time to travel (e.g. to a hospital), social representation or geographical concept [7-9]. The Organisation for Economic Co-operation and Development (OECD) defines rurality as areas with population densities below 150 inhabitants per square kilometer [10].

In Germany, the GP shortage is a growing problem, especially in some “rural” areas [11,12]. Our research group decided to work on the understanding of “rurality” in Germany as it is a country with a history of minimal access to care inequalities

Federal states in Western Germany have an average density of 264 inhabitants per square kilometer; while federal states in Eastern Germany have a density of 152 inhabitants per square kilometer [13]. However, from a German point of view there are rural areas in western federal states too, e.g. the western federal state of Baden Württemberg consists, according to the Ministry of Rural Area, Alimentation and Consumer Protection, of 70% rural areas [14]. From a health care point of view, the definition of rurality by density has a significant limitation as it does not indicate whether a practice is located in an area where there are difficulties in access to healthcare [15,16]. To support strategies to address the GP shortage in rural areas, our research group searched for an instrument measuring rurality from a health care delivery point of view. In Canada, a General Practice Rurality Index was introduced in 1997 including remoteness from closest basic/advanced referral centre, drawing population, number of GPs, number of specialists and presence of an acute-care hospital [17]. However, as the dimensions of rurality and the distances to the closest place of medical care between Canada and Germany were so extreme, a different existing instrument better applicable to the German setting was needed. In addition, from previous studies, we knew that young General Practice trainees are willing to drive up to 30 minutes to their place of work and that factors attracting them to work in a rural area were mainly related to the infrastructure of the locality [18]. Furthermore, from a health care delivery point of view, working with colleagues, minimal on-call duties and a hospital nearby have been shown to be important factors [18].

The New Zealand Rural Ranking Scale (RRS) had items meeting some of these demands of German trainees. The six items of the RRS are: travelling time from the surgery to major hospital, on-call duty, on-call for major traumas, travelling time to nearest GP colleague at place of work, travelling time to most distant practice boundary and regular peripheral clinic. The RRS was originally developed to identify GPs working in rural areas to pay them a bonus. GPs scoring 35 points or greater out of 100 are considered to work in rural practice in New Zealand [19].

The aim of this study was to translate the RRS, to culturally adapt it to the German health care delivery conditions, to validate it and to define cut-off scores for urban, semi-rural and rural areas in Germany.

## Methods

To translate and adapt the RRS instrument for German health care settings the Principles of Good Practice for the Translation and Cultural Adaptation Process by the ISPOR task force were considered [20]. The following steps were taken:

1. We asked for permission from one of the authors of the RRS to develop a German version and received permission in August 2010.

2. Two researchers separately conducted two independent forward translations.

3. The forward translations were discussed in the research group. Within this discussion process, three of the six items were changed and one was replaced due to differences in the health care systems between New Zealand and Germany.

4. The culturally adapted, translated instrument was piloted with three GPs to check for clarity of understanding and potential ambiguities. As four out of the six items were changed, a back translation step was not performed. However, results were discussed with the author of the original instrument.

To validate the instrument, in September 2011 the modified RRS-Germany (mRRS-G) was sent to 724 GPs located in urban (n = 250), semi-rural (n = 221) or rural areas (n = 253) in three different federal states. The definition of “urban”, “semi-rural” and “rural” was based on the definition used by the Federal Institute for Research on Building, Urban Affairs and Spatial Development [8]. These definitions are basically based on population densities the OECD uses. GPs in these regions were identified through lists of the local Associations of Statutory Health Insurance Physicians. Participants were asked to complete the six items of the mRRS-G and additional socio-demographic questions.

The ethics committee of the Heidelberg Medical School informed the research group previously that an ethic approval was not necessary.

Continuous data were summarized using means and standard deviations. Categorical data are presented as frequency counts and percentages. In accordance with the original RRS scale, a total score was calculated for the mRRS-G summing up the scores of the six variables namely: traveling time to next major hospital, on-call duty, receiving timely backup by a paramedic team, traveling time to nearest general practitioner colleague at place of work, traveling time to most distant practice boundary and satellite clinic. Cut-off points were calculated by means of the mRRS-G for the groups working in rural, semi-rural and urban practice. Group comparisons of rural, semi-rural and urban practice location regarding age of participants and mRRS-G score were done using ANOVA with Bonferroni correction for post-hoc tests and for gender with Chi^2^ test.

Furthermore, principal factor analysis was performed (eigenvalue > 1, varimax rotation) and the Kaiser-Meyer-Olkin (KMO) measure of sampling adequacy and the Bartlett’s test of sphericity was determined. Convergent construct validity was assessed in terms of Spearman rank correlation test between the means of each item of the mRRS-G score. Reliability was assessed using Cronbach’s alpha, which indicates whether an item of a scale is appropriate for assessing the underlying concept of its scale [21].

Predictors for rurality were calculated by binary logistic regression analysis. The following covariates were included in the regression analysis: distance to the next major hospital, on-call duty, receiving backup by a paramedic team, travelling time to nearest general practitioner colleague at place of work, travelling time to most distant practice boundary and satellite clinic. The analyses were performed using SPSS version 20.0 (SPSS Inc., Chicago IL, USA). An alpha level of P < 0.05 was used to test statistical significance.

## Results

### Translation process

All six items of the RRS were translated, three items were culturally adapted and one item had to be replaced by a new item.

The scale of the item “travelling time to next major hospital” was changed from the original “30 up to 90 minutes” to “up to 15”- “more than 91 minutes”. Also, the scale of the item “on-call duty” had to be changed from the original ranging from “one in one” up to “one in six” into categories ranging from on-call within 1–5 physicians up to “25 or more”. For the item “regular peripheral clinic” the aspect of traveling time was added.

The item “on-call for major traumas” had to be completely changed as family physicians in Germany usually have no on-call responsibility for major trauma. Within a consensus meeting, a new item was developed. The new item needed to meet the aspects of remoteness and, as in the original item, of emergency care but also represent a typical situation for GPs in Germany. For the new item, the question “In case of an emergency, do you receive backup by a paramedic team within 15 minutes?” was developed. The scale ranging from 0 to 15 came from the original scale. The score was “0” if the participant to date never had an emergency call-out, “5” if the backup by the paramedic team reaches the physician within 15 minutes and 15 if the backup by the paramedic team would reach the physician in later than 15 minutes. All changes were reported to the original author and appraised as “very appropriate” in terms of reflecting the different context of rural practice within the German system. The mRRS-G in English language can be found in the Additional file 1.

### Scores with the mRRS-G scale

Overall response rate was 34% (n = 244) with a 38% (n = 92) response rate from rural areas, 34% (n = 83) from semi-rural areas and 28% (n = 69) from urban. Average age of the participants was 54 years and 44% (n = 107) were female. Most participating GPs (54%) worked in a sole practice and had an average work experience level of 18.6 years. For more details see Table 1. There were no significant differences regarding the age of the rural, semi-rural and urban participants. However, gender among the semi-rural and urban participants was significantly different. Less female participants were from semi-rural areas (26.2% semi-rural versus 40.1% urban) whereas in urban areas more female doctors (38.3% urban versus 20.4% semi-rural) participated in the study.

**Table 1:** **Sociodemographic characteristics of the study sample (n = 244)**^**†**^

	**Mean (SD)**
Age, years	54 (8.2)
Experience as a GP, years	18.6 (9.2)
	Total number (percent)
Gender	
Male	137 (56.0)
Female	107 (44.0)
Mode of practice	
Single practice	132 (54.0)
Group practice	109 (45.0)
Location of the practice	
Rural	92 (37.7)
Semi-rural	83 (34.0)
Urban	69 (28.3)

^†^n varies due to missing data; SD: standard deviation.

**Figure 1:**
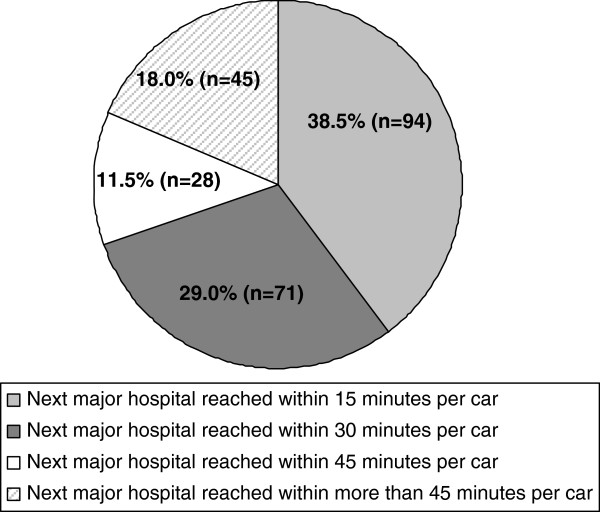
Time needed to reach next major hospital.

**Figure 2:**
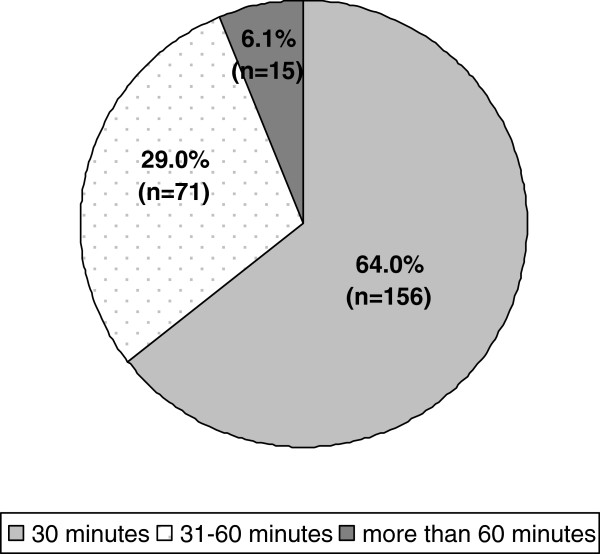
Maximum time needed to reach the most distant boundary covered by the practice.

**Figure 3:**
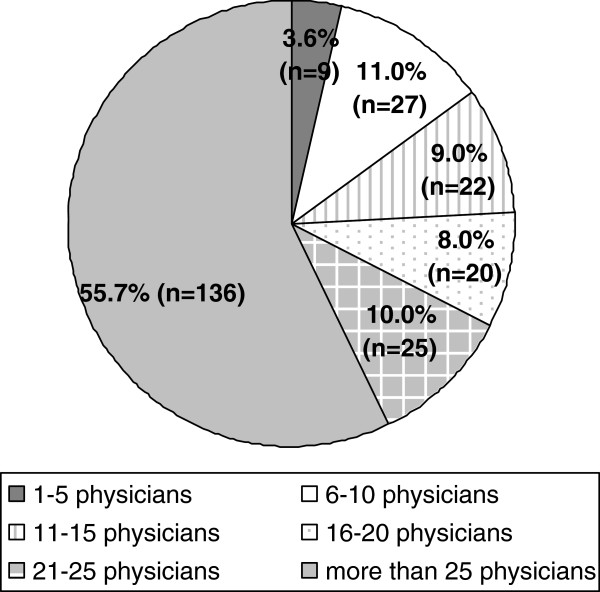
Number of physicians sharing on-call duties.

Most participants (67.5%) indicated that travelling time to the next major hospital was within 30 minutes with a mean of 22.3 minutes (SD: 22.3; min 0 to max 100). The mean number of physicians taking part in an on-call service was 8.8 (SD: 1.7). 90% (219) receive backup by a paramedic team within 15 minutes, whereas 8.2% (20) do not. 97% (237) can reach the next general practitioner colleague at place of work within 15 minutes by car. 64% needed a maximum of 30 minutes to reach the most distant boundary covered by the practice (one direction) and 88% (214) did not have a satellite clinic. For more details please see Figures 1, 2, 3.

Of the participants, 9 (4%) met the New Zealand cut-off point for rurality of 35 or more. The mean score from a practice located in a rural area was 16. Therefore, the cut-off point for rurality in Germany was defined as 16, for semi-rural areas as 13 and as 8 for urban areas. For more details please see Table 2.

**Table 2:** Cut-off scores

	**Mean score (SD)**	**95%CI**
Rural score	16 (8.9)	14.0-17.8
Semi-rural score	13 (8.6)	11.5-15.3
Urban score	8 (5.7)	7.0-9.8

CI: confidence interval; SD: standard deviation.

The ANOVA showed, that the differences of the mRRS-G mean scores between rural- and urban area as well as between semi-rural- and urban area were significant (p < 0.05). However, the difference between rural- and semi-rural areas was not significant.

The items “on-call duty”, “next major hospital” and “maximum time needed to reach the most distant boundary covered by the practice” were identified as significant predictors for rurality and showed a Nagelkerkes R^2^ of more than 17% (r^2^ ~ 0.172). The other covariates “receiving backup by a paramedic team”, “traveling time to nearest general practitioner colleague at place of work” and “satelite clinic” were not significant. For more details see Table 3.

**Table 3:** Predictors for rurality

	**OR**	**95%CI**	**p value**
on-call duty	1.03	1.01-1.04	<0.01
Travelling time to next major hospital	1.08	1.03-1.13	<0.01
Travelling time to most distant practice boundary	1.13	1.03-1.24	0.01

Nagelkerkes R^2^: 0.17; Hosmer-Lemeshow-Test: 0.15.

OR: odds ratio; CI: confidence interval.

Factor analysis revealed three dimensional structure of the mRRS-G scale with explained variance of R^2^ = 59.4% (KMO 0.493, Barlett’s test of spericity P = 0.001). The Spearman rank correlation coefficients showed low correlation between all six items. A negative correlation was observed between the item regarding the number of colleagues taking part in the on-call service and the other five items of mRRS-G scale. The Cronbach’s alpha of the mRRS-G scale was negative.

## Discussion

On average participants were 54 years old and 44% were female. These numbers reflect national German averages, where the age of physicians is 52 years and 42% being female. The RRS is applicable to the German health care setting, however, during the adaption process, the answer scales of three items had to be modified and one item had to be replaced completely due to health care system differences in the two countries. Explorative factor analysis showed that the mRRS-G has more than one dimension. Internal consistency showed a negative Cronbach’s alpha, indicating that the construct of rurality itself is more complex. Nevertheless from a health care delivery point of view, the mRRS-G can effectively define rural areas. The rurality cut-off point of 16 in the mRRS-G is far less than the New Zealand RRS which is 35 [19].

From a previous study [11], we concluded that the majority of patients in Germany can reach a hospital within 30 minutes, therefore, we changed the original scale in relation to this measure. Also, the scale of the item “on-call duty” had to be changed, as in Germany the density of physicians is higher than in New Zealand [22] and moreover, not only GPs but all physicians working in ambulatory care have to take over on-call duties.

The “on-call duty” item was one of the significant predictors for rurality. In the original RRS, the scale regarding the “on-call duty” item goes from 1–5 colleagues. In our sample, 136 from 244 physicians had more than 25 colleagues taking part in an on-call service. It can be assumed that the negative correlation of the “on-call duty” item between the other five items was responsible for the negative internal consistency. Naturally, areas with so many physicians sharing on-call duties cannot be labeled “rural” from a health care delivery point of view.

In Germany, there is an on-going debate as to how to attract more young physicians to work in rural areas. One important strategy might be getting own experience with rural practice during the undergraduate and postgraduate phases [23]. However, the optimal duration and type of contact with working/practicing in rural areas needed is quite unclear yet [24]. It seems for a country like Germany that the most important strategy would be offering young doctors the opportunity to experience that in practice there are no relevant differences between rural and non-rural areas from a health care delivery point of view [25]. This is very important because in Germany during medical school or postgraduate training, students/residents have almost no obligatory contact with rural areas.

The impression of young doctors as to what is rural from a health care delivery point of view might therefore be vague and influenced rather by subjective factors or just perceptions [18,26]. Common false perceptions about GPs working in rural group practices in Germany include e.g. that working in rural areas means more working hours and that there is less access to specialists [16,18,27].

The mRRS-G allows us to make the categorization of “rural” according to the local healthcare infrastructure and, therefore, provide evidence to counter some of the subjective perceptions of what this means. Finally, the mRRS-G might be a useful tool to identify areas where a) students or trainees could get experience with rural practice (rurality) in terms of health care delivery and b) to detect gaps in service provider numbers in the local health care system. Additionally, the mRRS-G could be - in accordance with the use in New Zealand - used as a basis for providing financial incentives for GPs working in rural areas.

Limitations of the study might be that a selection bias in favor of the more motivated physicians answering the questionnaire cannot be excluded.

As one item had to be completely replaced by an item exploring another aspect of emergency care, the instrument is not a purely adapted version, but a modified instrument.

Furthermore, as the correlations between the six items of mRRS-G were weak, a negative Cronbach’s alpha resulted. It could be assumed that the mRRS-G scale needs further examination in an additional, larger sample.

## Conclusion

This study is the first to introduce an instrument exploring the question of rurality from a health care delivery point of view in a country with traditionally little access to care inequalities. The mRRS-G is an easy to use six-item instrument. Although the cut-off point for rurality is low compared to New Zealand, it was possible to define “rural area” in Germany with the mRRS-G from a health care delivery point of view. Therefore, this instrument can be used to identify rural areas and provide data for specific strategies against GP shortages in Germany. False perceptions held by medical students and postgraduate trainees could also be addressed by using data produced from this instrument. Furthermore, this instrument could help GP stakeholders as well as communities to identify gaps in a local health care system and negotiate for measures to address unmet needs. Finally, although the mRRS-G has significant value, the construct of rurality itself cannot be limited to the definition in the mRRS-G. More research is needed to develop instruments catching more aspects of rurality.

## Competing interests

Authors declare that they have no competing interests.

## Authors’ contributions

JS, PO, KG, JSz and SJ have made substantial contributions to conception and design of the study, JS and PO translated the RRS and collected the data, analysis and interpretation of data was performed by KG and JS. JS, KG and PO have been involved in drafting the manuscript. SJ and JSz provided input improving the manuscript. All authors have given final approval of the version to be published.

## Pre-publication history

The pre-publication history for this paper can be accessed here:

http://www.biomedcentral.com/1472-6963/14/147/prepub

## Supplementary Material

Additional file 1 Modified Rural Ranking Scale-Germany (mRRS-G). Click here for file
