# Supplementary material for: Rural area in a European country from a health care point of view: an adaption of the Rural Ranking Scale
Source: BMC Health Serv Res. 2014 Apr 2;14:147. doi: 10.1186/1472-6963-14-147 (PMC4021561; doi:10.1186/1472-6963-14-147)
Supplement: Additional file 1 — Modified Rural Ranking Scale-Germany (mRRS-G). [file 1472-6963-14-147-S1.docx]

**Additional file 1**

**Appendix: Modified Rural Ranking Scale-Germany (mRRS-G)**

| 1. How many colleagues (yourself included) take part in general practitioners (after hour care) on-call duties in your area (if your city (region) has more than one general practitioners on call service, please indicate how many persons in total are on the after hour roster)? | | | | | |
| --- | --- | --- | --- | --- | --- |
| 1 | 2 | | 3 | 4 | 5 |
| 6-10 | 11-15 | | 16-20 | 21-25 | 25 or more |
| 2. In case of an emergency, do you receive backup by a paramedic team within 15 minutes? | | | | | |
| yes | | no | to date had not an emergency call-out | | |

*Please note that in the following questions travelling times refer to one-way journeys by car in normal daytime conditions and travelling within legal speed limits.*

| 3. Travelling time from your practice to next major hospital | | | | | | | | | | | | | |
| --- | --- | --- | --- | --- | --- | --- | --- | --- | --- | --- | --- | --- | --- |
| up to 15  minutes | 16-30  minutes | | | | 31-45  minutes | | 46-60  minutes | | | 61-90  minutes | | | more then 91 minutes |
| 4. Travelling time to nearest general practitioner colleague at place of work | | | | | | | | | | | | | |
| 0-15 minutes | | 16-60 minutes | | | | more but 61 minutes | | | | |  | | |
| 5. Travelling time to your satellite clinic(s) | | | | | | | | | | | | | |
| do not have any | | | | up to 30 minutes | | | | 31-60 minutes | | | | more then 60 minutes | |
| 6. Travelling time to most distant boundary covered by your practice | | | | | | | | | | | | | |
| up to 30 minutes | | | 31-60 minutes | | | | | | more then 60 minutes | | | | |
